# Mortality of the Child-bed as Affected by the Number of the Labor

**Published:** 1866-01

**Authors:** 


**Mortality of the Child-bed as affected by the Number of the Labor.**

The *Edinburgh Medical Journal* for September last contains an interesting paper on this subject by Dr. J. Matthews Duncan. The author, after presenting the statistics he has collected, remarks, "It must be noted that I have, hitherto, at least, said nothing regarding the nature of the relation between the number of the delivery and the mortality attending it. It is true the data recorded demonstrate more or less completely certain coincidences, which may be called laws. But they establish nothing further. These laws are as follows:

"1. The mortality of first labors is about twice the mortality of all subsequent labors taken together.

"2. The mortality from puerperal fever following first labors is about twice the mortality from puerperal fever following all subsequent labors taken together.

"3. As the number of a woman's labor increases above nine the risk of death following labor increases with the number.

"4. As the number of a woman's labor increases above nine, the risk of death from puerperal fever following labor increases with the number.

"5. If a woman has a large family, she escapes extraordinary risk in surviving her first labor, to come again into extraordinary risk as she bears her ninth and subsequent children.

"These laws, although they merely state coincidences, have very important practical bearings, which are too self-evident to require description. They have also important philosophical bearings, which were alluded to in the commencement of this article. The most important, perhaps, of these relate to puerperal fever. These also I shall not enter upon farther than to say, that the attendance of puerperal fever specially on primiparæ, and women who have born large families—its pretty close correspondence in relative amount to the general mortality of parturition after different pregnancies—its subjection also to the law of the duration of labor—do not appear to me to lend support to the views hitherto generally entertained regarding it, and expressed in the words accidental fever, contagious, epidemic. Another point under this head I shall merely mention. Authors, comparing the mortalities of lying-in institutions, whether from puerperal fever or from other causes, are frequently found neglecting to begin by ascertaining whether or not they are fit objects of comparison, and under this head, *inter alia*, neglecting to ascertain the comparative amount of primiparity in each institution. It is plain that, unless there be nearly the same comparative amount of primiparity in the institutions, their respective gross mortalities cannot be justly compared with one another."—*Am. Jour. Med. Sciences.*

